# A National Pension Fund for Hospital Officials and Nurses

**Published:** 1887-04-16

**Authors:** 


### A National Pension Fund for Hospital Officials and Nurses.

The Views of Officials, Sisters, Nurses, and Others.

### By a Private Nurse.

I have often thought I should like to tell you how very much I appreciate The Hospital, and how eagerly I look forward for it every week. I think it is a most valuable paper, and has supplied a great want in the nursing profession. The only wonder now is why such an organ of communication and knowledge was not thought of before. And it is not only interesting to the fraternity, but I find that wherever I go (and I am continually changing my habitation, as I am what is called a private nurse) the people are equally pleased with it. In fact, a gentleman I have nursed just lately is taking it himself, and says that it has opened up quite a new channel of thought for him. I may add that he passed through a very severe illness, and I think anything connected with nursing and the medical profession generally will now always appeal to his sympathy.

To go to the subject I am really writing about, viz., "The National Pension Fund for Hospital Officials and Nurses," now on the *tapis*, and which has been more forcibly brought to my mind by what a private nurse says in last week's Hospital, that she "believes many of the public who employ nurses would be pleased to give donations to such a fund in recognition of the nurse's services." I should like to say that this is my opinion also. I may add in support of this, that a very short time ago a family who employed a trained nurse sent £30 to the Institution to which I belong, and I am quite sure they would with many others be as willing if not more pleased to contribute to a National Pension Fund, from which the nurses would themselves receive benefit. I think it is a splendid idea, and one that should meet with approval and gratitude by all concerned. I have no doubt that the great majority of nurses (when the plan is laid out before them in detail) will be only too glad to avail themselves of such an opportunity of laying by for a "rainy day," which, if it does not come, is as well to be prepared for.

I think with Mr. Michelli that the scheme should be based upon principles of self-help, and everyone concerned encouraged to subscribe as much as they could afford annually. I feel confident that if we did make a determined effort to help ourselves as much as possible towards providing a pension fund, we should meet with plenty of support, as those who persevere in a just cause generally do. There are plenty of rich people in this country, and who prove themselves so in charitable deeds, who, I feel sure, would, if they were appealed to, come forth and help us, and I scarcely think they would ever regret so celebrating this remarkable year, the Queen's Jubilee, by giving pecuniary assistance to that part of the community who spend the best part of their lives in nursing and tending the sick and afflicted.

If you think my paper worth publishing, and I am not taking up too much of your valuable space, I would also add that there is a nurses' home in London that provides pensions for its nurses of £20 a year after twelve years' service in the same, the fund of which is supplied by an extra charge of a shilling a week being made for each nurse's services, together with all extra profits of the institution, and by donations, as nurses here are not allowed to receive presents. Should they leave before the expiration of the twelve years, through ill-health, or to be married, they receive sums of from £5 to £15, according to the length of time they have served, and so, providing they stay long enough, they really get all they earn. I merely mention this as showing what even one small institution of nurses can do towards providing for their future, as they did not number twenty when I knew them a few years ago.

---

### By Miss Waters, Matron of the Hampshire Nurses' Institution.

Two of our nurses are most interested in your proposed "National Pension Fund for Hospital Officials and Nurses." (Perhaps you would not wish to have the names of nurses from a *Private Institution* among it numbers.) I have no doubt that more of our nurses would join such a scheme, and I myself should be very glad to see it established, for I think it is, as you very justly say, "of vital importance to all," but my sympathy would be equally with the "Private" as with the "Hospital" nurse, for I consider their life in many ways more trying.

---

### By an Earnest Worker.

I am sure there are many nurses who would be glad to subscribe to an annuity fund if one was started. But as the nurses are such an *underpaid* class, often receiving much smaller salaries than domestic servants, it would be better if a good sum could be raised to start the fund with. Surely there are hundreds of people who have derived much benefit from being nursed by trained nurses, who would be glad to come forward and help such a deserving cause with liberal donations or yearly subscriptions. I think if it is done that it would be just as well *not* to have any hard and fast rule about candidates having a certificate. Why should such a thing be an essential, when there are *hundreds* of both private and hospital nurses without? Some good deserving women, *too*, who have been in the work for years, who have been obliged through family affairs or ill-health to get their training by degrees; some who, after their first year, have taken to private work, which is an equally useful branch of nursing. In fact, a woman who has had both hospital and private work makes the best nurse, for nursing single individuals in private houses teaches a nurse things she would *never* learn, even if she remained in a hospital for sixty years. And some of these things are *positively necessary* before a woman can be considered a good nurse. I would like to go into this question, but space will not permit, so I will only add that I hope the annuity fund will be started, and that it will be open to all those who have taken up the work, and intend to go on with it through life, keeping themselves wholly for the "Master's use."

